# Psychosocial profile of adults with Congenital Heart Disease according to documented history of anxiety disorders

**DOI:** 10.1371/journal.pone.0360616

**Published:** 2026-10-22

**Authors:** Efrén Martínez-Quintana, Marta Isabel García-Suárez, María Alcántara-Castellano, Jesús María González-Martín, Fayna Rodríguez-González

**Affiliations:** 1 Cardiology Service, Complejo Hospitalario Universitario Insular-Materno Infantil, Las Palmas de Gran Canaria, Spain; 2 Department of Medical and Surgical Sciences, Faculty of Health Sciences, Universidad de Las Palmas de Gran Canaria, Las Palmas de Gran Canaria, Spain; 3 Research Unit, Complejo Hospitalario Universitario Insular-Materno Infantil, Las Palmas de Gran Canaria, Spain; 4 Hospital Universitario de Gran Canaria Dr. Negrín, Las Palmas de Gran Canaria, Spain; Scuola Superiore Sant’Anna, ITALY

## Abstract

**Background:**

Adults with congenital heart disease (CHD) may experience substantial psychological distress. This study evaluated whether a documented history of anxiety disorders is associated with a distinct psychosocial profile in adults with CHD.

**Methods:**

In this cross-sectional study, 45 adults with CHD were included: 21 with a documented history of anxiety disorders and 24 without. Psychosocial assessment included the EuroQol-5D-3L (EQ-5D-3L), Rosenberg Self-Esteem Scale (RSES), State–Trait Anxiety Inventory (STAI), and Beck Depression Inventory-II (BDI-II). Associations between psychosocial measures and documented anxiety history were assessed using multivariable logistic regression adjusted for sex, NYHA functional class, and CHD complexity. Moderate depression was additionally assessed using univariable logistic regression.

**Results:**

Mean age was 35 ± 9 years in participants with a documented history of anxiety disorders and 31 ± 11 years in those without (*P* = 0.230); males accounted for 33% and 62%, respectively (*P* = 0.052). NYHA functional class II–IV was more frequent among participants with a documented history of anxiety disorders (67% vs. 25%; *P* = 0.018). Compared with those without a documented anxiety history, participants with anxiety history reported greater limitations in usual activities (*P* = 0.021), higher anxiety/depression scores on the EQ-5D-3L (*P* = 0.023), poorer self-rated health (*P* = 0.038), higher trait anxiety (31.2 ± 5.8 vs. 26.5 ± 5.2; *P* = 0.008), and greater depressive symptom severity (BDI-II median 19 vs. 3; *P* = 0.001). Self-esteem was also higher (RSES median 27 vs. 25; *P* = 0.006). After adjustment, BDI-II scores were positively associated with a documented history of anxiety disorders (OR 1.14, 95% CI 1.05–1.28; *P* = 0.011), while EQ-5D VAS scores were inversely associated (OR 0.64, 95% CI 0.38–0.97; *P* = 0.048). STAI-State, STAI-Trait, and RSES scores were not significantly associated with anxiety history. None of the variables assessed in the univariable analysis was significantly associated with moderate depression.

**Conclusions:**

In adults with CHD, a documented history of anxiety disorders was associated with greater depressive symptom severity and poorer self-rated health after adjustment for relevant clinical factors.

## 1. Introduction

Psychological disorders are highly prevalent among individuals with chronic illnesses and represent an important determinant of quality of life and clinical outcomes. Conditions such as diabetes, cardiovascular disease, and autoimmune disorders are frequently associated with sustained psychological distress, including anxiety, depression, and chronic stress. These difficulties may arise from the long-term nature of the disease, treatment burden, and uncertainty regarding prognosis, and may adversely affect symptom perception, coping behaviors, treatment adherence, and healthcare use [1].

Cardiovascular disease may impose not only physiological limitations but also substantial psychosocial challenges. Fear of adverse cardiovascular events, restrictions in physical activity, and the need for long-term medical follow-up may contribute to chronic stress, reduced perceived control, social withdrawal, and impaired interpersonal relationships [2, 3].

Congenital heart disease (CHD) comprises a heterogeneous group of structural cardiac abnormalities present at birth. Its reported incidence ranges from 4 to 50 per 1,000 live births [4, 5]. Advances in diagnosis, surgical treatment, and long-term care have enabled more than 90% of affected individuals to survive into adulthood [6]. Nevertheless, many adults with CHD require repeated interventions, implanted cardiac devices, or lifelong medical treatment and surveillance. In addition, functional and neurodevelopmental difficulties, including language impairments, attentional deficits, and behavioral problems, may contribute to the increased prevalence of anxiety and mood disorders reported in this population, affecting approximately one-third of adults with CHD [7–14]. These findings support the integration of psychological assessment into routine CHD care.

Despite the high prevalence of anxiety among adults with CHD, its identification and characterization in clinical practice remain heterogeneous. Most previous studies have relied on symptom-based psychometric instruments administered at a single point in time, whereas fewer have examined the clinical relevance of a previously documented history of anxiety recorded in the medical chart. In routine practice, psychiatric history is frequently considered during clinical decision-making and may provide complementary longitudinal information regarding psychological vulnerability. However, its interpretation is also influenced by access to mental healthcare, diagnostic practices, and the completeness of medical records. It therefore remains unclear whether adults with CHD and a documented history of anxiety exhibit a distinct psychosocial profile compared with those without such a history.

The aim of this study was to compare clinical characteristics, health-related quality of life, and psychological outcomes in adults with CHD according to the presence or absence of a documented history of anxiety. We hypothesized that patients with a documented history of anxiety would exhibit greater psychological symptom burden and poorer self-perceived health and functional status.

## 2. Methods

### 2.1. Study design and participants

This observational, analytical, cross-sectional study was conducted at a tertiary referral outpatient clinic specializing in CHD. Adult patients aged 18 years or older with a confirmed diagnosis of CHD were consecutively recruited during routine follow-up visits between January 1 and June 1, 2023.

The diagnosis of CHD was established through a comprehensive review of medical records, including previous imaging studies, such as echocardiography, cardiac magnetic resonance imaging, or computed tomography, together with surgical reports when available. CHD complexity was classified according to established clinical criteria [15].

Participants were stratified according to the presence or absence of a documented history of anxiety disorder recorded in the electronic medical record before study inclusion. Patients classified as cases had a documented diagnosis of an anxiety disorder, irrespective of current or previous pharmacological treatment. Controls had no documented history of anxiety disorder and no prior use of anxiolytic medication.

The diagnosis of anxiety had been established during routine clinical care by the treating healthcare professional and was not reassessed for the purposes of this study. Information regarding the healthcare professional responsible for the diagnosis (e.g., psychiatrist, psychologist, cardiologist, or general practitioner), the diagnostic criteria used (e.g., *Diagnostic and Statistical Manual of Mental Disorders* or *International Classification of Diseases*), and the exact date of diagnosis was not consistently available in the medical records. Consequently, these variables could not be included in the analyses. Anxiety symptoms reported during the study interview were recorded for descriptive purposes only and were not used to classify participants, thereby minimizing the risk of misclassification based on transient symptomatology.

Patients were excluded if they had cognitive or neurological disorders that could interfere with questionnaire completion; severe psychiatric disorders other than anxiety; an acute cardiovascular event (including myocardial infarction, cardiac surgery, or heart failure decompensation) within the previous six months; pregnancy; inability to complete the questionnaires because of language, visual, or comprehension barriers; severe comorbid conditions limiting life expectancy; incomplete medical records; or insufficient information to determine psychiatric history.

### 2.2. Data collection

Demographic, clinical, psychiatric, and socioeconomic information was collected through structured interviews and a review of the electronic medical records. Demographic variables included age and sex. Clinical variables comprised the type and complexity of CHD, New York Heart Association (NYHA) functional class, cardiovascular risk factors (systemic arterial hypertension, diabetes mellitus, dyslipidemia, and smoking status), the number of previous cardiac surgical procedures, and the presence of implantable cardiac devices, including pacemakers and implantable cardioverter-defibrillators.

Psychiatric variables included documented psychiatric diagnoses and the use of anxiolytic medication. Information regarding the timing of the anxiety diagnosis, comorbid depression, antidepressant treatment, and previous psychological or psychiatric care was not systematically available and therefore could not be incorporated into the analyses.

Socioeconomic variables included occupational status (employed, retired, homemaker, student, unemployed, or other) and educational level (primary education, secondary education/vocational training, or university education).

The study was conducted in accordance with the principles of the Declaration of Helsinki and was approved by the Research Ethics Committee (CEIm; approval code: 2022-033-1). Written informed consent was obtained from all participants before enrolment.

### 2.3. Psychosocial assessment

Psychosocial outcomes were evaluated using validated self-administered instruments assessing health-related quality of life, self-esteem, anxiety, and depressive symptoms. All questionnaires were completed during routine outpatient visits under standardized conditions.

Health-related quality of life was assessed using the EuroQol-5D-3L (EQ-5D-3L), which evaluates five domains: mobility, self-care, usual activities, pain/discomfort, and anxiety/depression. Each domain is scored across three levels of severity. Overall self-rated health was assessed using the EQ visual analogue scale (EQ-VAS). In accordance with the validated Spanish version used at our institution, participants rated their current health on a scale ranging from 0 (worst imaginable health state) to 10 (best imaginable health state) [16, 17].

Self-esteem was assessed using the Rosenberg Self-Esteem Scale (RSES), a 10-item questionnaire evaluating global self-worth. Each item is scored on a four-point Likert scale, with higher scores indicating greater self-esteem [18–20].

Anxiety was assessed using the State–Trait Anxiety Inventory (STAI), which distinguishes state anxiety, reflecting transient emotional responses, from trait anxiety, representing a relatively stable predisposition to perceive situations as threatening. Each subscale consists of 20 items scored on a four-point Likert scale. Raw STAI-State and STAI-Trait scores were analysed without age- or sex-standardization [21, 22].

Depressive symptoms were assessed using the Beck Depression Inventory-II (BDI-II), a 21-item questionnaire evaluating cognitive, affective, and somatic symptoms of depression. Each item is scored from 0 to 3, with higher total scores indicating greater depressive symptom severity. A BDI-II score >20 was selected to identify participants with at least moderate depressive symptoms according to the original instrument classification [23, 24].

All questionnaires have been validated in Spanish populations and are considered appropriate for adults with chronic diseases.

### 2.4. Outcomes

The primary objective of the study was to compare the psychosocial profile of adults with CHD according to the presence or absence of a documented history of anxiety disorders. The principal outcomes were trait anxiety, depressive symptom severity, self-esteem, and self-rated health. Secondary outcomes included state anxiety and each individual dimension of the EQ-5D-3L.

For exploratory analyses, depressive symptoms were additionally dichotomized using a BDI-II score >20 to identify participants with at least moderate depressive symptoms.

### 2.5. Statistical analysis

Continuous variables were summarized as mean ± standard deviation (SD) or median (interquartile range [IQR]), according to their distribution. Normality was assessed using the Shapiro–Wilk test. Categorical variables were presented as absolute and relative frequencies (percentages).

Between-group differences in continuous variables were assessed using Student’s *t* test for normally distributed variables and the Mann–Whitney *U* test for non-normally distributed variables. Categorical variables were compared using the chi-square test or Fisher’s exact test, as appropriate.

To assess the associations between psychological, health-related, and quality-of-life scale scores and case–control status, separate multivariable logistic regression models were fitted for each quantitative scale score. Case–control status was specified as the binary dependent variable, with cases considered the event. All models were adjusted a priori for sex, New York Heart Association (NYHA) functional class, and CHD complexity. For each scale score, the regression coefficient (β), standard error (SE), odds ratio (OR), 95% confidence interval (CI), and *P* value were estimated. Model fit was assessed using the Akaike information criterion (AIC), and discriminative performance was evaluated using the area under the receiver operating characteristic curve (AUC-ROC).

The association between moderate depression and age, sex, NYHA functional class, and CHD complexity was evaluated using logistic regression, with moderate depression specified as the binary outcome. Univariable logistic regression models were initially fitted for each candidate variable. A multivariable model including age, sex, NYHA functional class, and CHD complexity was subsequently fitted. Given the small sample size and the limited number of participants with moderate depression (12 of 45 participants), an additional parsimonious exploratory multivariable model including sex and NYHA functional class was fitted to reduce model complexity. This model was considered a sensitivity analysis and should be interpreted as exploratory. Odds ratios and 95% CIs were reported for all models. Model fit was assessed using the AIC, and discriminative performance was evaluated using the AUC-ROC.

All statistical tests were two-sided, and a *P* value <0.05 was considered statistically significant. Statistical analyses were performed using R software, version 4.6.1 (R Core Team, 2026).

## 3. Results

A total of 45 adults with CHD were included in the study, comprising 21 patients with a documented history of anxiety and 24 without such a history.

The spectrum of CHD was heterogeneous in both groups. Among controls, the most frequent diagnoses were tetralogy of Fallot, atrial septal defect, and coarctation of the aorta, whereas tetralogy of Fallot, atrioventricular septal defects, and transposition of the great arteries predominated among patients with a documented history of anxiety. Although severe CHD was numerically more common in the anxiety group (38.1% vs. 16.7%), no statistically significant differences in CHD complexity were observed between groups (p = 0.195).

Baseline demographic and clinical characteristics are summarized in Table 1. There were no significant differences in age, sex distribution, cardiovascular risk factors, previous cardiac surgery, or the presence of implantable cardiac devices. Occupational status and educational level were also comparable between groups. However, patients with a documented history of anxiety were significantly more likely to present with New York Heart Association (NYHA) functional class II–IV than controls (67% vs. 25%, p = 0.018).

**Table 1:** Baseline clinical characteristics and psychological outcomes according to documented history of anxiety.

Variable	Controls (n = 24)	Cases (n = 21)	p value
Sociodemographic characteristics			
Age, years	31 ± 11	35 ± 9	0.230
Male sex, n (%)	15 (63)	7 (33)	0.052
Educational level, n (%)			0.525
Literate (read and write)	1 (4)	1 (5)	
Primary education	2 (8)	1 (5)	
Secondary education / Vocational training	15 (63)	15 (71)	
University education	6 (25)	4 (19)	
Occupational status, n (%)			0.683
Employed	11 (46)	7 (33)	
Retired	1 (4)	3 (14)	
Homemaker	1 (4)	3 (14)	
Student	6 (25)	4 (19)	
Unemployed	3 (13)	1 (5)	
Other	2 (8)	3 (14)	
Clinical characteristics			
Moderate–severe CHD complexity, n (%)	15 (63)	17 (81)	0.195
NYHA class II–IV, n (%)	6 (25)	14 (67)	0.018
Hypertension, n (%)	5 (21)	2 (9)	0.422
Diabetes mellitus, n (%)	0 (0)	0 (0)	—
Dyslipidemia, n (%)	1 (4)	4 (19)	0.169
Current smoker, n (%)	0 (0)	1 (5)	0.467
Current anxiolytic medication, n (%)	0 (0)	6 (29)	0.007
Pacemaker, n (%)	1 (4)	2 (9)	0.592
Implantable cardioverter-defibrillator, n (%)	0 (0)	3 (14)	0.055
Number of cardiac surgeries	1.2 ± 1.4	1.9 ± 0.9	0.055
Quality of life (EQ-5D-3L)			
Mobility*	2 (8)	3 (14)	0.435
Personal care*	0 (0)	1 (5)	0.467
Usual activities*	3 (12)	9 (43)	0.021
Pain/discomfort*	5 (21)	8 (38)	0.433
Anxiety/depression*	8 (33)	15 (71)	0.023
EQ-5D VAS score	8.0 ± 1.7	6.8 ± 1.9	0.038
Psychological assessment			
Rosenberg Self-Esteem Scale	25 (24 –26)	27 (25 –29)	0.006
STAI-State	51.2 ± 6.3	51.1 ± 5.7	0.967
STAI-Trait	26.5 ± 5.2	31.2 ± 5.8	0.008
Beck Depression Inventory-II	3 (3–7.5)	19 (6–27)	0.001

Data are presented as mean ± standard deviation (SD), median (interquartile range), or n (%), as appropriate. Continuous variables were compared using Student’s t test or the Mann–Whitney U test, and categorical variables using the χ² test or Fisher’s exact test. Moderate or severe limitation reported in each EQ-5D dimension. * Values represent participants reporting any impairment (levels 2–3) in each EQ-5D-3L dimension.

Regarding health-related quality of life, patients with a documented history of anxiety reported significantly greater limitations in usual activities (43% vs. 12%, p = 0.021), a higher frequency of problems in the anxiety/depression dimension of the EQ-5D-3L (71% vs. 33%, p = 0.023), and poorer self-rated health as measured by the EQ-5D visual analogue scale (6.8 ± 1.9 vs. 8.0 ± 1.7, p = 0.038). No statistically significant differences were observed in the mobility, self-care, or pain/discomfort dimensions.

Psychological assessment revealed significantly higher trait anxiety among patients with a documented history of anxiety compared with controls (STAI-T: 31.2 ± 5.8 vs. 26.5 ± 5.2, p = 0.008), whereas state anxiety did not differ between groups (STAI-S: 51.1 ± 5.7 vs. 51.2 ± 6.3, p = 0.967). Depressive symptom severity was also substantially greater in the anxiety group (BDI-II median 19 [interquartile range, 6–27] vs. 3 [interquartile range, 3–7.5]; p = 0.001), corresponding to mild depressive symptoms among cases and minimal depressive symptoms among controls. Self-esteem scores were modestly but significantly higher in patients with a documented history of anxiety (median 27 vs. 25, p = 0.006), although both groups remained within the range generally considered to represent moderate self-esteem.

As shown in Table 2, after adjustment for sex, NYHA functional class, and CHD complexity, the Beck Depression Inventory-II score was positively associated with a documented history of anxiety disorders (OR 1.14, 95% CI 1.05–1.28; *P* = 0.011), indicating higher odds of having a documented history of anxiety disorders with increasing depression scores. In contrast, the STAI-State score was not significantly associated with a documented history of anxiety disorders (OR 0.96, 95% CI 0.83–1.09; *P* = 0.541). The STAI-Trait score (OR 1.16, 95% CI 1.00–1.38; *P* = 0.069) and the Rosenberg Self-Esteem Scale score (OR 1.35, 95% CI 1.00–1.94; *P* = 0.063) showed positive associations that did not reach statistical significance. The EQ-5D VAS score was inversely associated with a documented history of anxiety disorders (OR 0.64, 95% CI 0.38–0.97; *P* = 0.048), indicating lower odds of having a documented history of anxiety disorders with increasing health-related quality-of-life scores. Among the models evaluated, the model including the Beck Depression Inventory-II score showed the lowest AIC (48.57) and the highest AUC-ROC (0.90). The AUC-ROC values of the other models ranged from 0.81 to 0.84 (Table 2).

**Table 2:** Summary of adjusted logistic regression models for documented history of anxiety disorders.

Outcome	β	SE	OR	CI (95%)	p-valor	AIC	AUC ROC
STAI-State	−0.04	0.07	0.959	0.83 - 1.09	0.541	58.95	0.81
STAI-Trait	0.15	0.08	1.16	1 - 1.38	0.069	55.52	0.83
Beck Depression Inventory-II	0.13	0.05	1.14	1.05 - 1.28	0.011	48.57	0.90
Rosenberg Self-Esteem Scale	0.3	0.16	1.35	1 - 1.94	0.063	55.37	0.84
EQ-5D VAS score	−0.44	0.23	0.64	0.38 - 0.97	0.048	54.93	0.83

Logistic regression models were adjusted for sex, NYHA functional class, and congenital heart disease (CHD) complexity. Results are presented as regression coefficients (β), standard errors (SE), odds ratios (OR), 95% confidence intervals (CI), p-values, Akaike information criterion (AIC), and area under the receiver operating characteristic curve (AUC-ROC).

### Factors associated with moderate depression.

The association between moderate depression and age, sex, NYHA functional class, and CHD complexity was assessed using univariable logistic regression. None of the variables was significantly associated with moderate depression. Age (OR 0.97, 95% CI 0.91–1.04; *P* = 0.452), female sex (OR 2.41, 95% CI 0.63–10.49; *P* = 0.214), NYHA class II–IV (OR 2.16, 95% CI 0.57–8.71; *P* = 0.263), and moderate or severe CHD complexity (OR 1.12, 95% CI 0.22–6.41; *P* = 0.900, and OR 1.67, 95% CI 0.29–10.66; *P* = 0.570, respectively) showed no statistically significant associations.

Given the small number of events, an additional parsimonious exploratory multivariable model including sex and NYHA functional class was fitted based on their comparatively lower *P*-values in the univariable analyses. Neither female sex (OR 2.09, 95% CI 0.52–9.43; *P* = 0.309) nor NYHA class II–IV (OR 1.83, 95% CI 0.45–7.65; *P* = 0.396) was significantly associated with moderate depression. The model had an AIC of 55.86 and an AUC-ROC of 0.64.

## 4. Discussion

This study evaluated the psychosocial profile of adults with CHD according to the presence of a documented history of anxiety disorders, a clinically accessible marker that is routinely available during clinical care but remains underexplored in research settings. The findings indicate that patients with a documented history of anxiety disorders were characterized by greater depressive symptom burden, poorer self-rated health, more pronounced functional limitations, and higher unadjusted trait anxiety than patients without such a history, despite otherwise comparable demographic and clinical characteristics.

Although patients with a documented history of anxiety disorders presented with a worse NYHA functional class and showed numerical differences in other baseline clinical characteristics, multivariable analyses demonstrated that BDI-II scores remained positively associated with a documented history of anxiety disorders, while EQ-5D VAS scores were inversely associated after adjustment for sex, NYHA functional class, and CHD complexity. In contrast, the associations with trait anxiety and self-esteem were attenuated and no longer reached statistical significance. These findings suggest that the observed differences in depressive symptom burden and self-rated health were not fully explained by sex, functional class, or CHD complexity. Nevertheless, the possibility of residual confounding by unmeasured clinical or psychosocial factors cannot be excluded.

Our findings extend previous evidence demonstrating the high prevalence of psychological distress among adults with CHD [12] by showing that a documented history of anxiety disorders is associated with a distinct psychosocial profile. Unlike cross-sectional symptom assessments, a documented psychiatric history reflects longitudinal clinical information accumulated during routine care and may therefore provide complementary information regarding patients’ psychological vulnerability. At the same time, it is important to recognize that documented psychiatric history is influenced by access to mental healthcare, diagnostic practices, and the completeness of medical records. Consequently, it should not be regarded as a surrogate for standardized psychiatric assessment but rather as an additional source of clinically relevant information. Furthermore, the cross-sectional design precludes any inference regarding causality or the temporal relationship between anxiety history and psychosocial outcomes.

Functional status emerged as an important component of this psychosocial profile. Patients with a documented history of anxiety disorders were more likely to have a worse NYHA functional class and reported greater limitations in usual daily activities, findings that are consistent with previous studies identifying functional impairment as a major correlate of psychological distress in adults with CHD [12, 13, 25–27]. Interestingly, poorer self-rated health also remained independently associated with a documented history of anxiety disorders after multivariable adjustment. Together, these observations suggest that patients’ subjective perception of health may capture important aspects of psychological well-being that are not fully explained by anatomical disease complexity or conventional clinical measures. These findings reinforce the importance of incorporating patient-reported outcomes into the routine evaluation of adults with CHD.

The observed dissociation between trait and state anxiety provides additional insight into the psychological characteristics of this population. Patients with a documented history of anxiety disorders exhibited higher unadjusted trait anxiety, suggesting a relatively stable tendency to experience anxiety. However, this association was attenuated after adjustment for relevant clinical variables and was no longer statistically significant. By contrast, no differences were observed in state anxiety, which may reflect the greater sensitivity of state anxiety to the immediate context in which psychological assessment is performed, whereas trait anxiety represents a more enduring psychological characteristic [28–30].

An unexpected observation was the slightly higher self-esteem found among patients with a documented history of anxiety disorders. This finding should be interpreted cautiously. Previous studies have suggested that self-esteem may be influenced by coping strategies, resilience, and social support in individuals living with chronic diseases [31–35]. However, these factors were not assessed in the present study and therefore cannot explain the observed difference. Moreover, the absolute difference between groups was small and the association was no longer statistically significant after adjustment, suggesting that its clinical relevance remains uncertain. Confirmation in larger prospective studies is warranted before any firm conclusions can be drawn.

The exploratory analysis of moderate depression did not identify significant associations with age, sex, NYHA functional class, or CHD complexity in univariable analyses. Given the small number of participants with moderate depression, these findings should be interpreted cautiously and do not exclude clinically relevant associations. Rather, they highlight the limited statistical power available to investigate predictors of moderate depression in this cohort.

From a clinical perspective, the present findings support the incorporation of psychological history into the routine assessment of adults with CHD. A documented history of anxiety disorders may identify patients with greater depressive symptom burden, poorer perceived health, and more pronounced functional limitations, who may benefit from a more comprehensive psychological evaluation. Rather than replacing standardized psychological screening instruments, psychiatric history may complement symptom-based assessment by providing longitudinal information that is readily available during routine clinical care. However, the present findings should be considered hypothesis-generating and require confirmation in larger prospective cohorts. This approach may facilitate earlier recognition of vulnerable patients and support more individualized multidisciplinary management. More broadly, our findings reinforce the importance of a holistic, patient-centred model of care in adults with CHD, in which psychological assessment complements conventional clinical evaluation.

Future studies should confirm these findings in larger and more diverse cohorts using prospective longitudinal designs capable of clarifying the temporal relationships between anxiety history, psychological vulnerability, and clinical outcomes. Further research should also evaluate the contribution of potentially modifiable factors, including coping strategies, resilience, social support, access to psychological care, psychiatric treatment, and socioeconomic determinants, to better understand the mechanisms underlying psychological distress in adults with CHD and to identify potential targets for intervention.

### 4.1. Limitations

This study has several limitations that should be considered when interpreting the findings. First, the relatively small sample size may have limited statistical power, particularly for multivariable analyses, and may reduce the generalizability of the results. The small number of participants with moderate depression further limited the ability to investigate potential predictors of this outcome. In addition, the cross-sectional design precludes causal inference and does not allow differentiation between pre-existing psychological vulnerability and disease-related adaptation. Furthermore, psychological variables were assessed using self-report instruments and may therefore be subject to reporting bias.

Second, anxiety classification was based on previously documented clinical diagnoses recorded in the electronic medical record rather than on standardized diagnostic interviews performed specifically for the study. Consequently, some patients with clinically relevant anxiety symptoms may have remained undiagnosed and been classified as controls, while variability in routine clinical diagnostic practices may also have influenced case ascertainment. In addition, information regarding the timing of the anxiety diagnosis, the healthcare professional establishing the diagnosis, and the diagnostic criteria used was not consistently available in the medical records. Such non-differential misclassification could have attenuated the observed associations.

Third, information regarding comorbid depression, antidepressant treatment, and previous psychological or psychiatric care was not systematically collected. Therefore, the higher BDI-II scores observed among patients with a documented history of anxiety disorders may reflect broader psychological distress, including coexisting depressive symptoms, rather than anxiety alone. Future studies should systematically assess psychiatric comorbidities and mental health treatments to better characterize the relationship between anxiety and psychological outcomes in adults with CHD. Likewise, other potentially relevant psychosocial factors, such as coping strategies, resilience, and social support, were not systematically assessed.

Finally, the exploratory logistic regression analysis of moderate depression was based on only 12 participants with moderate depressive symptoms. Consequently, multivariable modelling was limited by the small number of events and was susceptible to imprecision and overfitting. None of the variables assessed in the univariable analyses showed a statistically significant association with moderate depression. These findings should therefore be interpreted as exploratory and hypothesis-generating rather than confirmatory. In addition, the heterogeneity of CHD may limit the applicability of these findings to specific patient subgroups.

## 5. Conclusions

In adults with CHD, a documented history of anxiety disorders was associated with a distinct psychosocial profile characterized by greater depressive symptom burden, poorer self-rated health, and more pronounced functional limitations. After adjustment for sex, NYHA functional class, and CHD complexity, depressive symptom severity and self-rated health remained associated with documented anxiety history, whereas associations with trait anxiety and self-esteem were attenuated. Although these findings require confirmation in larger prospective studies, documenting a history of anxiety may help clinicians recognize patients who could benefit from further psychological assessment and individualized multidisciplinary care.
